# Experimental and Computational Nanotoxicology—Complementary Approaches for Nanomaterial Hazard Assessment

**DOI:** 10.3390/nano12081346

**Published:** 2022-04-14

**Authors:** Valérie Forest

**Affiliations:** Mines Saint-Etienne, Univ Lyon, Univ Jean Monnet, Etablissement Français du Sang, INSERM, U1059 Sainbiose, Centre CIS, F-42023 Saint-Etienne, France; vforest@emse.fr

**Keywords:** nanomaterials, toxicity, experimental models, computational models, in vitro, in vivo, in silico

## Abstract

The growing development and applications of nanomaterials lead to an increasing release of these materials in the environment. The adverse effects they may elicit on ecosystems or human health are not always fully characterized. Such potential toxicity must be carefully assessed with the underlying mechanisms elucidated. To that purpose, different approaches can be used. First, experimental toxicology consisting of conducting in vitro or in vivo experiments (including clinical studies) can be used to evaluate the nanomaterial hazard. It can rely on variable models (more or less complex), allowing the investigation of different biological endpoints. The respective advantages and limitations of in vitro and in vivo models are discussed as well as some issues associated with experimental nanotoxicology. Perspectives of future developments in the field are also proposed. Second, computational nanotoxicology, i.e., in silico approaches, can be used to predict nanomaterial toxicity. In this context, we describe the general principles, advantages, and limitations especially of quantitative structure–activity relationship (QSAR) models and grouping/read-across approaches. The aim of this review is to provide an overview of these different approaches based on examples and highlight their complementarity.

## 1. Introduction

In addition to ultra-fine particles produced naturally (as by-products of wildfires, volcanic eruptions, and other natural processes) or unintentionally released as a consequence of human activities (present in polluting emissions, such as welding fumes, cigarette smoke, aircraft waste gas, or diesel exhaust), engineered nanomaterials are produced purposefully to take advantage of their unique physical, chemical, and/or biological characteristics associated with their nanostructure. The remarkable development and applications of nanomaterials lead to an increasing release of these materials in the environment, and, consequently, ecosystems and humans are increasingly exposed to them, while the adverse effects they may elicit are not always fully characterized. By January 2022, the Nanodatabase [1] counted 5224 products containing nanoparticles available on the European market.

Besides the huge amount and variety of nanomaterials, we should keep in mind that nanomaterials are highly heterogeneous in terms of physicochemical features, which represents a challenge for their hazard assessment and the generalization of conclusions [2,3]. As a result, a thorough characterization of nanomaterials is a prerequisite to the evaluation of their potential toxicity [4,5]. In particular, the ISO/TR 13014:2012 guideline [6] recommends the systematic consideration of parameters such as particle size and particle size distribution, aggregation/agglomeration state, shape, surface area, composition, surface chemistry, surface charge, and solubility/dispersibility.

The toxicity of nanomaterials should be carefully assessed as a prerequisite for their safe and successful use in any application. To that purpose, many models and approaches are available, each having their advantages and limitations. The nanotoxicology field was first introduced in 2005 by Oberdörster et al. [7] and has evolved ever since. It is now also referred to as “nanosafety” [8] and includes in vitro and in vivo studies, and it aims to establish a dose/effect relationship and thresholds below which no biological effects are observed. It also studies the influence of specific properties of nanomaterials on biological responses; this interdisciplinary approach involves physicochemists as well as biologists. Furthermore, nanotoxicology includes the investigation of the interactions of nanomaterials with living organisms and the study of the mechanisms of action potentially responsible for adverse effects. Although nanotoxicology is a specific field due to the specific nature of nanomaterials, some methodologies and approaches can be derived from standard toxicology. Indeed, the toxicity of metals and other elements either naturally occurring or produced by anthropogenic activities has already been extensively studied in the context of environmental pollution [9].

We can roughly distinguish two types of approaches to evaluate nanomaterial hazards: (i) empirical toxicology consisting of conducting in vitro or in vivo experiments and (ii) in silico approaches relying on computational studies. The aim of this review is twofold: (i) to provide an overview of these different fields based on recent examples of applications and (ii) highlight their complementarity. Indeed, while the literature is rich in reviews focused either on experimental or computational approaches (sometimes involving slightly different scientific communities), very few studies deal with both. Thus, the present paper aims to bridge this gap by exploring a balanced combination of wet and dry lab approaches that are available for the toxicity assessment of nanomaterials, arguing for cooperative projects.

## 2. Experimental Nanotoxicology/Empirical Approaches

Experimental toxicology includes in vitro and in vivo studies. Basically, the principle is to expose individuals, tissues, or cells to the substance of study, observe the effects induced, and compare them with the response observed in a control group (treated in the same conditions but unexposed to the substance). Clinical studies can also be considered as part of experimental nanotoxicology.

The main parameters used to evaluate nanotoxicity include the study of the targets of the nanomaterials (biodistribution and cellular uptake), the assessment of the induction of cytotoxicity (cell damage, cell death), the pro-inflammatory response, oxidative stress, and genotoxic effects as these events have been reported to be the mechanisms of action of nanomaterial toxicity [10,11,12].

As mentioned before, a prerequisite to the assessment of the toxicity of nanomaterials is their thorough physicochemical characterization as some features can deeply influence the biological outcomes [7,13,14].

### 2.1. In Vitro Studies

#### 2.1.1. In Vitro Models

A wide variety of in vitro models exist: from cell lines to primary cultures (developed from tissue biopsies) and tissues. Primary cultures are quite challenging to establish due to the lack of tissue availability, the specific handling they require, and donor-specific variations [13,15]. Usually, cells lines are preferred because of their homogeneity and stability, resulting in reproducible results. However, they are either cancer cells or cells artificially immortalized, and although their high proliferative rate makes them easily cultivable, available in large quantities, and inexpensive, they exhibit altered pathways compared to normal cells. Consequently, they do not answer exactly as healthy cells would do, making them a poorly reliable representation of what really occurs in vivo. In addition, if they are used for long periods of time, cell de-differentiation and thus a change in phenotype can occur [13,15]. This is why some scientists question their relevance. However, Verdon et al. observed a similar pattern of response between the human neutrophil-like HL-60 cell line and human primary neutrophils after exposure to various nanomaterials [16]. They concluded that although some neutrophil functions were compromised in the cell line, it still represented a valuable model that can be used in a first attempt, especially for screening purposes, and primary cells can be used for more focused assessments.

Additionally, different cell types can be chosen depending on the scientific question to address: cells from the immune system, in particular macrophages, epithelial cells, fibroblasts, cells from the lung, cells from the gastrointestinal tract, neurons, etc. For instance, while fibroblasts are commonly used to assess pro-fibrotic signals, macrophages are used because they are the first line of defense of the organism. Additionally, lung and gastrointestinal cells are often used as they represent the primary target organs after the inhalation and ingestion of nanomaterials, two major exposure pathways [13]. 

Cell lines also offer the possibility to work with different species. While human cell lines are supposed to more closely mimic human responses in comparison to rodent cell lines, the latter are particularly useful for comparison of the results with animal data [13].

It should be taken into account that cell type-dependent effects have been reported, and this is why it is recommended to use several cell types to assess nanomaterial toxicity before drawing firm conclusions [15].

Cell culture conditions should also be considered carefully as they can impact the reproducibility and accuracy of the results. Drasler et al. reviewed and made recommendations on the main experimental parameters involved in in vitro assays to achieve a reliable assessment of nanomaterial–cell response [13]. For instance, the concentration of nanomaterials used in such experiments should be realistic and range from 1 to 100 μg/mL, with the lower and higher limits at 0.125 and 200 μg/mL, respectively. It is also recommended to include negative and positive controls to allow comparison between studies, both intra- and interlaboratory [13].

The use of cell lines in monoculture systems is very common and is indeed recommended for the first stage of nanomaterial hazard evaluation for the sake of simplicity [13]. However, it is acknowledged that they do not reproduce a tissue or organ which possesses a defined three-dimensional (3D) structure and do not include the complex cross-talks between cells [13,14,15,17,18,19]. To overcome these shortcomings, models able to more closely mimic a physiological reality have been developed. These models of increasing complexity are referred to as advanced in vitro models and include co-cultures and 3D cultures [18,20].

Multiple-cell cultures, also known as multicellular systems, 3D models, or “oid” cultures (e.g., organoids, spheroids), represent more physiologically relevant systems, especially for mechanistic studies, and have been described to have the potential to be more predictive in toxicology testing, thus filling a gap between 2D systems and animal experiments [8,13]. On the other hand, advanced culture systems are more expensive, time-consuming, technically more demanding, more difficult to standardize, and usually less suitable for high-throughput analyses [18,21].

Co-culture systems can be more or less complex; they comprise two or more cell types and are not necessarily 3D. For example, in the study of the effects induced by inhaled nanomaterials, co-cultures of alveolar cells and macrophages are interesting as the latter cell type can provide information on an indirect toxicity mechanism where nanomaterial internalization by macrophages leads to an inflammatory response and subsequent tissue damage [14,17]. In an attempt to simulate the alveolar epithelial barrier, Barosova et al. [22] developed a human alveolar cell co-culture model using alveolar epithelial type II cells and two types of immune cells (human monocyte-derived macrophages and dendritic cells). Another type of 3D lung co-culture model was developed to assess the hazard potential of multi-walled carbon nanotubes, consisting of a mix of alveolar epithelial cells, fibroblasts, and macrophages [23].

Similarly, different models of the 3D gastrointestinal tract were developed to assess the hazard of nanomaterials [24,25,26]. While Saez-Tenorio et al. used a Caco-2/HT29 co-culture, Bredeck et al. used a triple culture consisting of Caco-2, HT29-MTX-E12, and THP-1 cells [24,26].

Usually, a higher sensitivity of co-culture models has been reported compared to that of monocultures. As an example, after exposure to nanomaterials, biological adverse effects were observed in A549+THP-1 co-cultures but not in A549 monocultures [27,28,29]. 

Multicellular spheroids and organoids even more closely resemble in vivo organs [14,15,30,31]. These tissues have enhanced morphologies and show increased functional activity [17]. The difference between spheroid and organoid lies in the fact that in spheroids, cells are free to form aggregates into a tight 3D ball, while in organoids, cells are embedded in a matrix and confer a more ordered configuration, serving as a scaffolding support [19].

Such advanced 3D models have been developed to mimic the liver, pancreas, or lung [31,32,33,34]. For instance, Kabadi et al. developed 3D lung microtissues using human lung epithelial cells, fibroblasts, and macrophages [32]. Upon exposure to multi-walled carbon nanotubes and asbestos fibers, they observed responses similar to those previously observed in vivo in a rodent model, validating this advanced in vitro model.

Further, the incorporation of tissues or organs on a microfluidic platform, so-called organ-on-a-chip, enables the evaluation of nanomaterial toxicity in highly dynamic conditions in vitro [35,36]. A lung-on-a-chip based on human vascular endothelial cells and alveolar epithelial cells has recently been developed for the assessment of the pulmonary toxicity of TiO_2_ and ZnO nanoparticles [37]. The same model was used to evaluate the pulmonary effect of air pollutant fine particulate matter [38].

Ready-to-use commercial solutions are also available such as Epithelix, EpiAirway, MatTek, and MucilAir. They are 3D human reconstructed epithelia of the respiratory tract. These cultures are made of primary airway epithelial cells isolated from different parts of the respiratory tract of healthy donors or patients with chronic lung diseases, asthma, or chronic obstructive pulmonary disease. However, these models are expensive, and the donor variation has to be taken into account [39,40,41,42]. 

Besides more physiologically relevant cell models, progress has been made to more realistically reproduce cell or organ exposure to nanomaterials. For instance, when considering airborne nanomaterials, air–liquid interface (ALI) systems better recapitulate the physiological exposure of the lungs and seem better suited than conventional submerged exposure assays to predict lung toxicity [40,43]. Indeed, when exposed to ceria and titania nanoparticles, human A549 lung epithelial cells and differentiated THP-1 macrophages cells were more sensitive at the ALI compared to under classical submerged conditions [43]. Similarly, Loret et al. reported that the biological responses of A549/THP-1 cells exposed to TiO_2_ and CeO_2_ nanoparticles were usually observed at lower doses at the ALI than in submerged conditions [27]. Others have also found a higher sensitivity of cells exposed at the ALI compared to submerged exposure experiments [41,44,45]. Therefore, exposure of cells at the air–liquid interface has been shown to be a valid and sensitive method to assess the toxicity of several poorly soluble nanomaterials in monocultures and co-cultures and could bridge the gap between traditional 2D in vitro assays and animal models of airway exposure [4,27,39,44,46,47,48,49,50,51]. 

#### 2.1.2. Main Biological Endpoints Considered

Nanomaterials can induce toxicity both in vitro and in vivo through various mechanisms such as oxidative stress, cell death mechanisms (apoptosis, autophagy, and necrosis), genotoxicity, and immunological responses [10,11,12,19,52]. Different assays allow evaluating these biological endpoints to report on the nanotoxicity; they can be subdivided into cytotoxicity assessment (including cell viability, proliferation, and cell death assays), pro-inflammatory response, oxidative stress, and genotoxicity [11]. To draw firm conclusions, a battery of tests should be employed, with each assay being considered depending on the cell type and nanomaterials used [13].

Cytotoxicity can be measured based on the observation of cells’ morphological alteration, measurement of cells’ viability, or the ability of cells to proliferate and form colonies. Cell viability assays are generally based on the use of colorimetric or fluorimetric assays [13], e.g., MTT, XTT, WST-1, Alamar blue, neutral red uptake, or evaluation of membrane integrity (LDH, trypan blue assays), whereas cell division or proliferation can be evaluated through clonogenic assays or cell counting, respectively. Moreover, apoptosis assays (e.g., TUNEL assay, caspase activation) or apoptosis/necrosis (Annexin V/PI) assays can be used to characterize the type of cell death induced by nanomaterials [10,11,52,53,54,55,56].

A widely used approach to assess the pro-inflammatory response consists of the analysis of the soluble factors such as cytokines or chemokines secreted by cells and assessed in the cell culture supernatant by an enzyme-linked immunosorbent assay (ELISA) [13].

Oxidative stress is acknowledged to be a major mechanism of nanomaterial toxicity [11]. Indeed, exposure to nanomaterials leads to the production of reactive oxygen species (ROS) and reactive nitrogen species (RNS) [11]. Cells generate ROS to maintain normal metabolism/homeostasis, but their overproduction can interfere with a variety of signal transduction pathways and even induce cell apoptosis, pro-inflammation, and DNA damage [5,11,13,56]. ROS can be detected using electron paramagnetic resonance (EPR) or fluorescent probes [11].

Genotoxicity describes the potential damage induced by nanomaterials to the DNA [13]. Primary genotoxicity can be distinguished from secondary genotoxicity: primary genotoxicity is defined as genetic damage induced by nanomaterials themselves, whereas in secondary genotoxicity, nanomaterials do not interact directly with the target cell but produce an inflammatory response in neighboring cells, resulting in the oxidative damage of DNA by ROS. Please note that primary genotoxicity can also be mediated by ROS formation formed in cells by the interaction of nanomaterials, e.g., with mitochondria [13,57]. To assess genotoxicity, different markers can be used evaluating DNA damage, gene mutations, and chromosomal damage [21]. They include the in vitro mammalian cell gene mutation assay and the in vitro mammalian cell micronucleus assay. The latter determines the chromosome breakage leading to the formation of an additional nucleus (micronucleus) during cellular division [13,33]. The micronucleus assay also assesses chromosomal loss, i.e., aneugenic effects. In addition, the comet assay is widely used to detect the genotoxic potential of nanomaterials, allowing the detection of single- and double-stranded DNA breaks in individual cells [11,58,59]. Other genotoxicity assays include γ-H2AX foci formation as a marker for DNA double-strand breaks but with limited mechanistic insight for investigations of more precise mechanisms of genotoxicity [13].

#### 2.1.3. Advantages of In Vitro Approaches

In vitro models offer many advantages. They permit different levels of study: organ, tissue, cell (one or several populations); they allow large screening of effects with a very small amount of test material; and they are very well adapted for the study of mechanisms, mainly for short-term studies [53,60]. As they are performed under controlled testing conditions, they allow a reduction in variability between experiments [53]. They were initially developed to apply the “3R” rule introduced in 1959 and aiming to Reduce, Refine, and Replace animal experiments [61], which is now widely encouraged by international legislation [21]. In 2010, the European Commission requested the partial and even full replacement of animal studies [18,62]. In addition to preventing ethical issues related to animal testing, such methods are easier, faster, and cheaper [11]. 

In particular, in nanotoxicology, in vitro models allow a better understanding of nanomaterial uptake by cells and translocation through biological barriers, cytotoxicity and cellular effects, and genotoxicity induced by nanomaterials in regard to their physicochemical features. Thus, such in vitro studies have allowed evidencing the involvement of a specific physicochemical parameter in the toxicity of some nanomaterials. For instance, correlations were observed between nanomaterial toxicity and their shape (cerium nanoparticles) [63], their surface charge (silica nanoparticles) [64,65], their particle size and surface chemistry (silicon carbide nanoparticles) [66,67], their agglomeration size (boehmite nanoparticles) [68], and their size, shape, and agglomeration (TiO_2_ nanoparticles) [28].

This type of data is very useful, especially in the context of safer-by-design approaches. Indeed, when the physicochemical parameters responsible for the toxicity of a nanomaterial are identified, we can alter them at the early stages of the nanomaterial development to produce safer materials [14]. 

#### 2.1.4. Limitations of In Vitro Approaches

Although very convenient, in vitro models have the major disadvantage of being too simplistic. They do not reflect the complexity of a whole organism and do not reproduce the toxicokinetics. Therefore, they may not be predictive of what really occurs in vivo. In addition, chronic effects cannot be tested with these models due to difficulties of performing long-term simulation [14,20,55,56,60]. This is why, despite its limitation due to ethical reasons, the use of animal models still remains necessary.

### 2.2. In Vivo Models

The main advantage of in vivo models is that they are able to better mimic physiopathological processes and allow the systemic evaluation of the effects triggered by nanomaterials. They also enable studying the defense mechanisms that could counterbalance a biological response (e.g., antioxidant defense, tissue repair mechanism, clearance by cells from the immune system). Indeed, whole organisms remain the most scientifically relevant models as they are able to capture effects of nanomaterials after they have entered the body and have been distributed and processed [69]. As a matter of fact, a major advantage of in vivo models is that they allow the assessment of the kinetics of nanomaterials through absorption, distribution, metabolism, and excretion (ADME) [14]. In addition, while the long-term effects of inhaled nanomaterials, for instance, cannot be studied in vitro, they can be observed in vivo as they are retained in the body for longer periods [60]. Finally, in vivo models could allow establishing modes of action of nanomaterials as well as studies on the second generation, for instance, in the case of reproductive toxicity assays [70,71] or embryotoxicity evaluation [72]. Another asset of in vivo models is that they allow considering the biopersistence of nanomaterials in the organism. This is of paramount importance in the sense that it is correlated with toxicity. Indeed, the most persistent particles can accumulate in tissues where they can elicit adverse effects [73]. For instance, compared to silver ions, silver nanoparticles were shown to be more persistent in the circulating system and organs in terms of overall body distribution [72].

In the nanotoxicity field, the most frequently used in vivo models are mammal models, in particular small rodents such as mice, rats, and rabbits because of their close resemblance to humans. In addition, they are cheaper and easier to be maintained than larger animals such as pigs, which are genetically very close to humans [11,14,15]. Dogs and primates show the highest similarity to the human respiratory system; however, they are rarely used for toxicity studies due to ethical issues and experimental costs. On the other hand, guinea pigs have been used for sensitization to inhaled antigens since their airways show similar sensitivity to mediators to human airways, while rodent lungs are less sensitive [60].

To assess nanomaterial toxicity in vivo, different parameters can be evaluated, including the biodistribution of the nanomaterials in the organism (localization in tissues or organs), clearance (examination of excretion and metabolism of nanomaterials at various time points after exposure), hematology, serum chemistry (examination of changes), and histopathology (evaluation of alterations and potential damage to tissues) [11,14,15,19]. The LC_50_ (concentration of nanomaterial that causes the death of 50% of the population), LOEC (lowest concentration that causes a noticeable effect on the organism), and NOEC (maximum concentration at which no effect is observed on the organism) can also be determined [14,19]. The genotoxicity of nanomaterials has also been widely investigated in rodent models with the added value of evaluating the response of a whole organism upon nanomaterial exposure. In addition, with an appropriate study design, several endpoints can be analyzed in a single animal, reducing the number of animals and the costs of the in vivo experiments. For instance, the in vivo genotoxic potential of nanofibrillated cellulose, TiO_2__,_ SiO_2_, or Al_2_O_3_ nanomaterials administered to mice was evaluated using several genotoxicity endpoints [74,75,76,77].

Regarding the exposure of animals to nanomaterials, different routes are possible such as inhalation, intravenous or intraperitoneal injections, ingestion, intratracheal instillation, dermal administration, or gavage, to be chosen depending on the question to address [14,60]. Many studies have focused on inhalation as it is a main exposure route. Whole-body exposure can be performed by placing animals in inhalation chambers. While it best corresponds to pulmonary exposure, it is difficult to perform because usually nanomaterials are contained in dry powder and thus need to be dispersed in airflow, which can generate high concentrations of nanomaterials and thus aggregation [60]. Moreover, it is challenging to precisely quantify the inhaled dose as nanomaterials are not only inhaled but also ingested (through licking of the coat where they are also deposited). In addition, the animals can avoid exposure by huddling together or burying their noses in corners of cages or in the fur of another animal [60]. The “nose/head-only” system has been developed to prevent some of these drawbacks where animals are immobilized in a tube, and only the nose or head is exposed to the source of nanomaterials. However, such an immobilization is stressful for the animals and can therefore produce biological impact, thus inducing biased results when monitoring biological parameters. Inhalation studies require specialized equipment and are more difficult and expensive to carry out than oral administration [69].

Another widely used exposure pathway of animals to nanomaterials is through intratracheal instillation [60,69]. Nanomaterials are suspended in a physiological solution which is deposited directly in the respiratory tract through a cannula inserted in the trachea. It is easy to perform and allows controlling the dose effectively inserted into the lungs, but this technique can cause local tissue damage and an uneven distribution of the test substance in the lungs [60]. Moreover, it is not a physiological exposure, and when nanomaterials are suspended in a liquid, a more or less homogeneous solution is produced, resulting in a lack of reproducibility of the experiments. 

The exposure route should be chosen depending on the availability of materials (whole-body exposure requires high amounts of material), the technical expertise of the personnel (intratracheal instillation is technically challenging), and the exposure duration [60]. Anyway, the exposure route should be considered carefully as it has been shown to have a significant impact on the results. As an example, inhaled single-walled carbon nanotubes in mice elicited a greater effect than instilled particles [78], while the opposite was found for titanium dioxide nanoparticles [79]. However, Warheit et al. observed similar results with both exposure routes [80]. 

The high doses tested and route of administration used in inhalation studies are not always relevant to human exposure scenarios and can result in so-called “overload” of the test system. This is why short-term in vivo inhalation studies (STIS) have been developed to reduce the need for 90-day inhalation studies [69].

Intravenous injection of nanomaterials is also commonly used, especially for the study of toxicity mechanisms, to mimic translocation through the blood barrier. Similarly, other pathways such as oral administration or skin contact also have their pros and cons and should be considered depending on the scientific question to address.

Besides the ethical reasons and legislations [62] that tend to limit animal testing to that which is strictly necessary, in vivo models have intrinsic limitations. As mentioned before, from a practical point of view, such models are expensive, and facilities and personnel are necessary for animal housing, feeding, care, etc., with increasing needs with the increasing size of the animal model. Experiments with animals can be long and complex to handle. Additionally, due to the huge amount and variety of nanomaterials, the systematic assessment of their toxicity profile in vivo is simply unfeasible and unsustainable [81]. In addition, while they remain the gold standard for toxicology testing, questions remain on the relevance of in vivo models when assessing hazard implications towards human health [8]. Indeed, because of anatomical, physiological, and biochemical differences between species, one may wonder if in vivo approaches are really predictive for humans [18,60,82]. As a matter of fact, some models may also not be sensitive enough. For instance, it has been reported that little pleural reactivity was induced in rats even with particles known to be very carcinogenic (e.g., asbestos). This is due to the rat lifespan being too short to assess diseases that can occur 30 to 40 years after exposure in humans [60]. Similarly, the drug thalidomide was shown to be inert within rodents, while it induced significantly detrimental effects on human fetuses [8].

### 2.3. Clinical Studies

Biological monitoring or biomonitoring is defined as “the repeated, controlled measurement of chemical or biological markers in fluids, tissues, or other accessible samples from subjects exposed or exposed in the past or to be exposed to chemical, physical or biological risk factors in the workplace and/or the general environment” [83]. 

**Figure 1:**
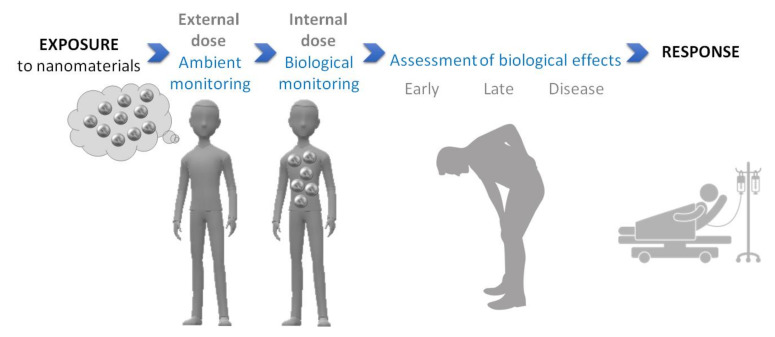
Biological monitoring of nanomaterials in human samples could fill a gap and help better understand the relationship between exposure to nanomaterials and adverse effects through the analysis of both biomarkers of exposure and biomarkers of effects.

Biomonitoring has been widely used in pulmonology, especially in the case of pneumoconiosis, and has uncovered critical information on the relationship between exposure to a harmful substance and biological and even pathological effects. For instance, the assessment of asbestosis bodies in patient lung tissues or in broncho-alveolar lavage (BAL) fluids has allowed defining values specific to diseases [84,85,86]. It has also been shown that BAL from patients suffering from idiopathic pulmonary fibrosis had a different chemical composition to that of patients with other interstitial lung diseases or healthy subjects [87]. In the context of health risk assessment, the biomonitoring of nanoparticles in human biological samples could be a particularly interesting approach to gain new insights into the role of inhaled biopersistent nanoparticles in the etiology/development of some respiratory diseases. Although technically challenging [88,89], this strategy appears promising. Indeed, the biomonitoring of nanoparticles in human lung tissues or fluids could fill a gap between exposure to nanomaterials (evaluated by the external dose assessed by ambient monitoring) and the biological effects and even diseases induced by these nanomaterials [88,90,91,92,93] (Figure 1).

We adopted this approach to detect and quantify nanoparticles in various types of clinical samples such as seminal and follicular fluids [94], the colon [95], amniotic fluids [96], or BAL [97,98,99]. We especially focused our attention on BAL, where we separated micron-sized particles (>1 µm) from submicron (100 nm − 1 µm) and nano-sized particles (<100 nm) contained in BAL from a cohort of 100 patients who suffered from interstitial lung diseases (ILDs). We then determined the metal load in each of these size fractions and evidenced a higher concentration of submicron silica particles in patients suffering from sarcoidosis than in patients suffering from other ILDs, suggesting a potential role of these inhaled particles in the etiology and/or development of sarcoidosis [98]. Similarly, we observed a higher concentration of titanium nanoparticles in patients suffering from idiopathic fibrosis than in patients suffering from other ILDs, allowing us to suspect a relationship between titanium nanoparticles and idiopathic pulmonary fibrosis even though, in this case, we had a limited number of patients to reach a satisfactory statistical power to draw firm conclusions.

We recently went one step further to gain a comprehensive vision of the events from exposure to airborne nanoparticles to the biological response induced (Figure 1) and investigated associations between respiratory diseases and occupational exposures [100]. To that purpose, we estimated the exposure to inhaled unintentionally released nanoparticles of patients for each job held in their working life. Most of the patients showed a high probability of exposure to airborne unintentionally released nanoparticles (>50%), suggesting a potential role of inhaled nanoparticles in lung physiopathology. Depending on the respiratory disease, the number of patients likely exposed to unintentionally released nanoparticles was variable (e.g., from 88% for idiopathic pulmonary fibrosis to 54% for sarcoidosis). These findings were consistent with the mineralogical analyses. Further investigations are necessary to draw firm conclusions, but these first results strengthen the array of presumptions on the contribution of some inhaled particles (from nano to submicron size) to some idiopathic lung diseases.

While BAL reflects a diluted sample of the respiratory tract lining fluid, it is invasive, takes time, must be performed in a medical setting, and cannot easily be repeated in short intervals. This is why exhaled breath, which is more easily accessible, has emerged as a promising matrix for biological monitoring [101]. The exhaled air is cooled to condensate, and the obtained exhaled breath condensate (EBC) is collected and analyzed for the presence of nanoparticles. 

Finally, of course, large epidemiological studies can be informative, but they are personnel- and time-consuming and require important financial resources.

### 2.4. Issues Associated with Experimental Nanotoxicology

#### 2.4.1. Lack of Standardized Assays

As described before, a wide variety of assays are available to assess nanomaterial toxicity [10]. However, there are no standardized protocols, and because of the different sensitivities of different cell lines, the influence of cell culture media, and dispersion techniques of nanomaterials, the outcome of in vitro assays may vary, making it difficult to compare results from different studies [14,56,102]. As an example, when reviewing studies from the literature to gain insight into the toxicity of graphene-based materials with regard to their physicochemical features, no firm conclusions could be drawn because of the lack of standardization of toxicity assessment as well as the incomplete nanomaterial physicochemical characterization [103]. There is also a lack of consensus on the dose metric; often, the concentration is expressed as mass per volume, which is not always the most relevant unit [15]. Indeed, the nanoparticle surface area or the nanoparticle number can be used as alternative dose metrics to nanoparticle mass. The more adequate metric should be chosen depending on the question to address; for instance, Oberdörster et al. demonstrated that to evaluate dose–response relationships of nanoparticle-induced pulmonary inflammation, the particle surface area was a more appropriate dose metric than particle mass or particle number [104]. Similarly, the biological endpoints can be expressed using different scales; for instance, cytotoxicity can be expressed using a binary system (toxic/nontoxic), or a (semi)quantitative scale (low/medium/high toxicity), with absolute values, LC_50_, NOAEL, LOAEL, etc. This heterogeneity makes it challenging to compare studies. 

Finally, failure to use standardized methods and appropriate control experiments questions the reliability of nanotoxicological assay results. In particular, the lack of positive controls at the nanoscale is an issue [105,106].

#### 2.4.2. Potential Artifacts

It has been well documented that nanomaterials can interact with different cytotoxicological assays, resulting in inaccurate results and misinterpretations [13,15,53,56]. In particular, due to their large surface area, nanomaterials can adsorb assay reagents, enzymes, substrates, or reporter dyes, inducing a bias in the assay outcome [15,107]. 

Moreover, as many cytotoxicity assays are based on optical readouts (spectrophotometric or fluorometric), the intrinsic optical properties of the nanomaterials (high absorption or scattering) can interfere with the detection method [15,53,107].

This is why it is highly recommended to evaluate possible interferences prior to any cytotoxicity assay to ensure reliable results. In case of interferences, nanomaterial-specific adaptations of the conventional cytotoxicity assays (e.g., MTT and DCF assays) might eliminate nanomaterial interference with the method [13,107]. Alternatively, after quantification and modeling of the interferences, corrective factors can be applied to obtain accurate results [108]. 

Kroll et al. characterized the interferences of 24 nanomaterials with 4 standard cytotoxicity assays for oxidative stress, cell viability, cell death, and inflammatory cytokine production (DCF, MTT, LDH, and IL-8 ELISA) and reported concentration-, particle-, and assay-specific interferences [107]. Similarly, it was demonstrated that interferences occurred between carbon nanotubes and the commonly used LDH assay, due to the carbon nanotubes’ intrinsic absorbance and their ability to adsorb LDH at their surface [109].

Besides such interferences, biological artifacts can also occur and bias the results. An example is the contamination by endotoxins or lipopolysaccharides (LPSs) that cause acute inflammatory responses in humans [55].

Another important factor inducing variation in nanotoxicity data is the medium in which the nanomaterials are dispersed. This can alter the agglomeration/aggregation state of the nanomaterial, which will subsequently have an impact on the cell uptake and toxicity of the nanomaterial [15]. This is why it is recommended to characterize the physicochemical properties of nanomaterials after dispersion in a cell culture medium. 

Consequently, possible nanomaterial–assay interactions should be carefully assessed. Furthermore, each biological endpoint should be evaluated with multiple assays which ideally supply complementary information and have different assay and detection principles, to validate the obtained results [15].

#### 2.4.3. Difficulty to Compare In Vitro and In Vivo Data

While the merit of in vitro models has been acknowledged, concerns remain about their correlation with in vivo data [60,102]. All the more, there is a lack of studies specifically dedicated to the comparison between results obtained from in vitro and in vivo approaches [69]. The in vitro/in vivo gap is an issue that makes the formulation of unambiguous conclusions on nanomaterial toxicity challenging [15].

Some factors can explain the conflicting results that have been observed between in vitro and in vivo data. First, as discussed before, in vitro models, especially standard 2D cell cultures, are too simplistic and do not recapitulate the complexity of whole organisms [15]. The development of advanced in vitro models will hopefully enable reducing this gap. Second, the nanomaterial doses used in vitro and in vivo may significantly differ due to the contribution of clearance [13] or due to the concentrations selected for the different assays. Indeed, while in vitro studies may use unrealistically high nanomaterial concentrations to highlight dose-dependent effects, such dosages would not be applicable in in vivo studies [102]. Third, we have to keep in mind that physicochemical features of the nanomaterials can deeply affect their toxicity, and even small changes in these features can lead to major alterations in nanomaterials’ interactions with biological systems [14,15]. 

However, efforts have been made to fill this gap; for instance, a rat NR8383 alveolar macrophage assay has been developed to assess the in vitro effects of SiO_2_ nanomaterials. The results were consistent with the effects observed in vivo, after intratracheal instillation of the nanoparticles in rat lungs [110]. Similarly, Kampfer et al. investigated the cytotoxicity profile of silver and titanium dioxide nanoparticles in four in vitro models of increasing complexity. The results were consistent with the in vivo effects of the same nanoparticles through tissue analysis from 28-day oral exposure studies in mice, especially for advanced in vitro models, underlining the relevance of these models [111].

#### 2.4.4. Formation of a Protein Corona in Biological Media

To fully explore the nanomaterial interactions with biological systems, we should take into account not only the “synthetic identity” of the nanomaterials (corresponding to their pristine, intrinsic nature), but also their “biological identity”, acquired in biological media [14,112]. Indeed, upon contact with blood, biological fluids, or cell culture media, biomolecules contained in these fluids adsorb on the nanomaterial surface, forming a crown, a layer mainly composed of proteins, the so-called “protein corona”. This new interface with biological systems will deeply impact the nanomaterial biological fate and response [113,114,115,116,117]. The composition of this protein corona is unique for each nanomaterial and influenced by the nanomaterial’s intrinsic physicochemical features as well as by biological environmental factors. The protein corona is also characterized by its dynamic nature [14,114,117]. Indeed, its composition can vary over time following what is called the “Vroman effect” where the most abundant proteins adsorb first and are then replaced by proteins of higher affinity [118]. The protein corona composition also varies, especially in vivo, depending on its location in the organism as the nanomaterial passes from one biological compartment to another, each having its own distinct set of proteins that interact in a unique way with the nanomaterial [117].

The characterization of the protein corona may be technically challenging, and a lack of reproducibility can also be mentioned. In addition, despite numerous works, no general rule can be established. Indeed, there is no universal corona; it is unique to each system as its formation depends both on the nanomaterial properties and the biological environment [65,117,119,120].

However, it is of paramount importance to take this component into account as it can deeply influence interactions with cells and the subsequent biological outcomes. Lesniak et al. showed that for identical particles and cells, under identical conditions, the interactions with cells and the biological outcomes could vary greatly in the presence or absence of a preformed corona in serum [121]. The protein corona may also impact the biopersistence of nanomaterials in vivo as it can be recognized by macrophages, resulting in an accelerated clearance [122].

Better knowledge of the impact of the protein corona is critical for nanotoxicology.

#### 2.4.5. Combined Effects of Nanoparticles

We are constantly exposed to numerous and various sources of nanoparticles (natural as well as engineered). It is thus difficult to reproduce such complexity in experiments; therefore, samples used in nanotoxicology assessment are not always fully representative of real-life exposure and may not lead to accurate human health risk assessment and management as they overlook the interactions between the contaminants and their resulting combined toxicity [123]. Indeed, the biological effects triggered by nanoparticles are usually assessed focusing on individual nanoparticles [124,125], while their interaction with co-contaminants can deeply impact, either positively or negatively, their biodistribution, fate in the organism, and toxicological profile (additive, synergistic, or antagonistic responses) [123,126]. Even if the toxicities of individual compounds are well characterized, unexpected adverse effects can be induced by the mixing of such compounds [127,128]. Even worse, compounds that do not elicit adverse effects individually can induce significant toxicity when in a mixture, resulting in an underestimation of the risk [129]. While additive or synergistic effects can occur (effects higher than the simple addition of the effects of the individual components) [130], antagonistic effects can also be reported [124,127,131,132].

Different mechanisms can be responsible for combined toxicity [123]. A carrier effect can be observed where the co-pollutant adsorbs at the surface of the nanomaterial that serves as a cargo and eases the entry and accumulation of the co-pollutant inside the cell. Moreover, the co-pollutant may alter the physicochemical features of nanoparticles, especially in relation to surface properties (chemistry, charge, hydrophobicity, agglomeration state, etc.), and consequently result in a modified toxicological profile. In addition, the first pollutant can make cells more sensitive to the second one. As an example, it was shown that ZnO nanoparticles altered cell membranes upon accumulation and therefore enhanced the toxicity of co-exposed Cu nanoparticles [133].

Despite the complexity of the issue (there are countless potential mixtures in our environment) and the technical difficulty to recapitulate real-life conditions, it seems crucial to take into account mixture effects [123]. Furthermore, if nanomaterial exposure is usually well considered during the production and manufacturing stages, it is less systematically evaluated during the use and end-of-life stages [81,112].

#### 2.4.6. Low Doses/Chronic Exposure

In the nanotoxicology field, most studies focus on acute exposure with high nanomaterial doses, while very few studies report on long-term exposures over weeks or months to sub-chronic exposures [13]. While long-term and repeated low-concentration exposure studies are scarce, they are extremely important as they better mimic real-life exposure [134]. Furthermore, the mechanisms involved in a biological response to a high single dose may be significantly different from those related to a repeated low-dose exposure [13]. Whereas in vitro studies can be carried out to evaluate acute effects, they are not adapted to long-term studies and chronic effects [13,135]. Such effects can be rather observed using in vivo models, even if they are time-consuming and expensive and few inhalation laboratories are equipped to carry out sub-chronic (90 days) or chronic (1.5–2 years) inhalation tests. This explains why the in vivo chronic effects of inhaled nanomaterials are poorly documented [69].

### 2.5. Perspectives and Future Developments for Experimental Nanotoxicology

Besides the development of advanced in vitro models that more closely mimic in vivo conditions, progress is awaited from the development of some approaches, techniques, and their combination. As examples, we can name the omics techniques, high-throughput screening (HTS) analyses, and the adverse outcome pathways (AOPs). 

The “omics” approaches include genomics, transcriptomics, proteomics, metabolomics, lipidomics, and toxicogenomics and can be applied both in vitro and in vivo (although more easily in vitro) [56]. They provide a more comprehensive understanding of the biological response to nanomaterials than conventional toxicological assays, providing an insight into the underlying mechanisms such as molecular interactions, cellular responses, tissue/organ changes, and organism responses [56,136,137,138,139]. In addition, they may predict toxicity at low levels of nanomaterial exposure, which do not produce toxicity but can stress the cells, thereby emphasizing subtle biological changes that would not be detected by a conventional toxicological assessment [56,139]. On the other hand, they require expensive infrastructure and highly skilled personnel to prepare the samples and to analyze the data [2]. 

High-throughput screening (HTS) methods are defined as the use of automated tools to facilitate the rapid execution of a large number and variety of biological assays that may include several test substances in each assay [2,21]. They thus allow the rapid screening of large numbers of nanomaterials at different concentrations and under different exposure conditions, saving time and money. In vitro HTS may be used in order to rank nanomaterials’ hazard potential and prioritize them for in vivo testing [2,112]. Several HTS methods have been validated and applied for nanomaterial testing, including high-throughput flow cytometry, multiplex analysis of secreted products, and genotoxicity methods (high-throughput comet assay, high-throughput micronucleus assay, and γH2AX assay) [2]. HTS can also be combined with high-content analysis (HCA) or high-content imaging (HCI) to deliver rapid and reliable toxicity assessment of large numbers of nanomaterials in parallel and can combine several endpoint measurements in one experiment [140]. For instance, the human hepatoma HepaRG cell line was treated with a large set of nanomaterials, coatings, and supernatants at different concentrations, and 14 different biological endpoints (including viable cell count, cell membrane permeability, apoptotic cell death, mitochondrial membrane potential, and steatosis) were analyzed using HTS [140]. The HTS method was also used to assess the genotoxicity induced by different graphene-based materials [57] or to evaluate the impact of nanomaterials on different DNA repair pathways [141].

An adverse outcome pathway (AOP) is a systematic process that uses the available mechanistic information concerning a toxicological response and describes causal or mechanistic relationships between a molecular initiating event (MIE), a series of intermediate key events (KEs), and the adverse outcome (AO) [56,142]. In other words, an AOP represents a linear chain of events starting from an MIE and ending with an AO. MIEs and AOs are connected by KEs corresponding to specific biochemical mechanisms which are causal and describe toxicity responses at different levels of biological organization including the cellular, organ, organism, and population levels [8,56,134,142]. The AOP framework provides pragmatic insights to promote the development of alternative testing strategies and can significantly support risk assessment by developing predictive methods that utilize mechanistic and evidence-based data [134,142]. The AOP approach has been applied to nanomaterials. While MIEs are not fully understood yet, inflammation, oxidative stress, and cytotoxicity have been identified as KEs and have led to defining lung fibrosis, lung emphysema, and lung cancer as AOs [56,134,142]. Different AOPs can be of relevance to nanomaterials such as AOP 173, AOP 303, AOP 237, AOP 302, and AOP 1.25, whose AOs are lung fibrosis, lung cancer, atherosclerotic plaque progression, acute inhalation toxicity, and lung emphysema, respectively [142]. However, lung fibrosis is the most widely assessed and reported AO following exposure to nanomaterials. AOPs can also be combined as networks. Indeed, individual linear AOPs allow the simplification of the complex biology, but the combination of AOPs could better describe the intricateness of the disease processes and hence is applicable to real-world scenarios [142]. 

Because of the respective drawbacks of each approach and model as discussed before, it is very difficult to draw firm conclusions on the hazard potential of nanomaterials. In addition, due to the multitude and variety of nanomaterials, it is impossible to assess in vitro or in vivo their toxicity profile on a case-by-case basis [143]. This is why computational approaches (in silico models) have been developed to complement experimental testing and represent useful alternatives proposing models able to predict the potential hazard of nanomaterials, without testing, thereby reducing the time and cost of nanosafety assessments and allowing the prioritization of nanomaterials that deserve further investigations [18,143].

## 3. Computational Nanotoxicology/In Silico Approaches

In silico toxicology refers to the use of computational methods to analyze, simulate, visualize, or predict the toxicity of chemicals and nanomaterials [54,144]. To be accurately predictive of nanomaterial toxicity, models should carefully consider the complexity of nanomaterials (requiring a complete physicochemical characterization) and their diverse environments (exposure route) [143,145]. Concretely, the aim of computational approaches is to model the relationships between nanomaterials’ structure, properties, and biological effects [143]. Therefore, reliable in silico models can be used for the supplementation of data in the first step of nanomaterial hazard assessment, as recommended by the European Chemicals Agency (ECHA), or assist in the second step of hazard assessment (categorization and labeling of nanomaterials) by directly classifying nanomaterials into groups of different hazards [146].

The main advantage of computational approaches is that they can reduce experimental (including animal) testing, thus saving time and money. They are fast and allow the screening of larger numbers of nanomaterials [147]. On the other hand, the development of in silico models has to face some challenges; in particular, to be robust, predictive, and broadly applicable, models should be developed based on large amounts of high-quality and complete experimental data. They have to take into account the wide heterogeneity of published literature data in terms of nanomaterial characterization, exposure, and hazard data reported [103,143]. Missing values or data gaps and differing data quality (numbers of replicates, signal-to-noise ratio, relevance of endpoints, different experimental conditions, etc.) can be a hurdle for modeling. 

Despite these limitations, different types of models of variable complexity have been successfully developed to predict nanomaterial toxicity. They mainly use statistical and machine learning (ML) algorithms to model relationships between a nanomaterial’s structure, molecular properties, or other parameters and its biological effects [143]. The main machine learning algorithms most commonly used include regression, decision trees, support vector machines, artificial neural networks, partial least squares, and principal component analysis [144,148,149]. Molecular dynamics (MD) has also been used to simulate and predict nanomaterial interactions with biological environments [150].

### 3.1. Molecular Docking

Molecular docking studies consist of computational simulations that allow predicting how nanomaterials interact with biomolecules based on 3D structural knowledge [20]. Molecular docking has also been used to determine the potential toxicity of CuO, TiO_2_, Fe_3_O_4_, Au, Ag, ZnO, Mn_2_O_3_, and Fe_3_O_4_ nanoparticles with biological macromolecules [151].

### 3.2. Quantitative Structure–Activity Relationship (QSAR)

**Figure 2:**
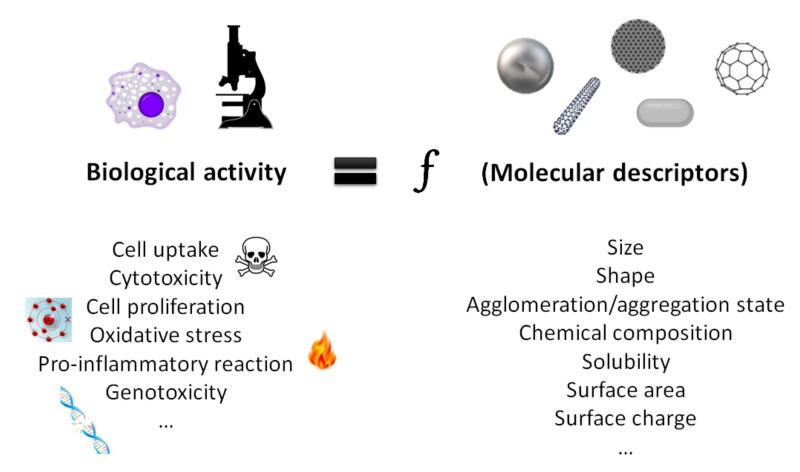
Basic principle of QSAR models for nanomaterials.

The most successful computational models able to predict the biological properties of nanomaterials in diverse and complex environments are based on the quantitative structure–activity relationship (QSAR) method [143]. The basic aim of the QSAR model is to define an appropriate function that has a reasonable relationship between the chemical structure and biological activity. This has the potential to further summarize the physio-chemical and biological information in order to predict toxicity effects or develop ideal nanomaterials [20]. In other words, and as schematically summarized in Figure 2, QSAR models are based on defining mathematical dependencies between the biological activity of nanomaterials (or toxicity, i.e., the endpoint) and their molecular descriptors (such as physicochemical features) [20,148,152]. They are usually used for a series of often related compounds: datasets.

This approach assumes that nanomaterials with similar molecular structures will have similar biological effects [153]. Consequently, such models are able to predict a nanomaterial’s behavior and effects in biological environments based on its physicochemical properties and molecular descriptors without experimental testing, which allows saving time and money [56,148,154]. Such predictions could also permit researchers to streamline and prioritize toxicological assays and focus on the most promising (i.e., safer) nanomaterials [114,154,155,156,157]. 

**Figure 3:**
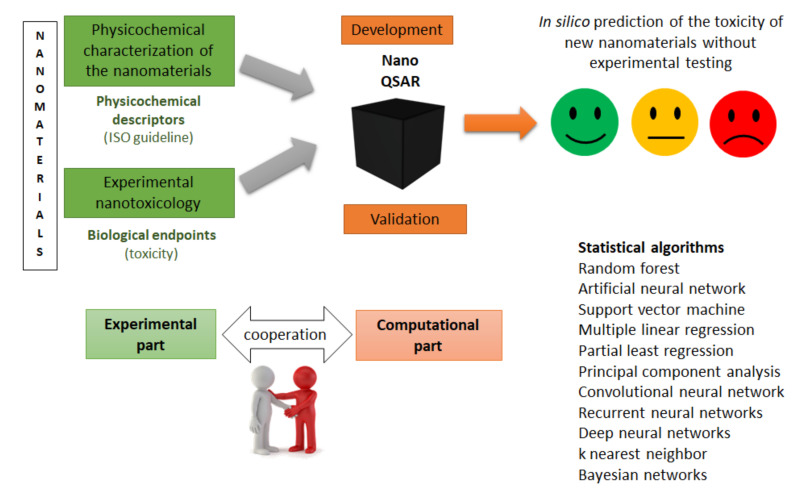
The development of QSAR models for the prediction of nanomaterial toxicity can use various statistical algorithms and is based on nanodescriptors (physicochemical features of nanomaterials) and biological endpoints (toxicity), both experimentally determined. This approach argues for strong collaboration between experimenters and modelers.

The concept of QSAR was proposed a century ago for chemical compounds [158,159] and has been extensively used in the areas of drug discovery and chemical toxicity modeling, but its application to nanomaterials is not without challenges due to their specificities. Indeed, it requires the development of nano-specific descriptors and curated experimental datasets [147,150,153,154,160]. As an example, a molecular descriptor can be experimentally determined or calculated [148]. In the latter case, we should ensure that the calculated descriptors are correlated with experimental values; otherwise, they could have a strong impact on the validity of the model. Therefore, descriptors should be developed and tailored using the material’s specific information provided by the physicochemical characterization (experimentally determined), rather than using idealized structures [154,160]. Regarding the datasets necessary to develop QSAR models, they should contain a sufficient number of diverse nanomaterials. These two conditions (diversity and a sufficiently high number of samples) are currently difficult to meet, except when HTS methods are used [154]. Indeed, there are only small databases on experimentally measured basic endpoints [161]. This is partly due to the fact that, although many nanotoxicological studies are available, they are not standardized, resulting in heterogeneous data, and the few nanomaterial databases available are often not suitable for building models [161]. In addition, nanomaterials represent very structurally diverse groups of chemicals, making it difficult to build a significant dataset of structurally related nanomaterials [152]. To sum up, a large quantity of experimental data of a high quality should be available to develop a reliable nanoQSAR model [150,152,156,162,163,164,165,166]. Stronger collaborations between experimentalists and modelers should result in more useful datasets and allow QSAR predictive models to improve their potential [154,161,167], as shown in Figure 3.

As recommended by the OECD [168], five principles should be met to validate a QSAR model: (i) it should be associated with a defined endpoint, (ii) show an unambiguous algorithm, (iii) a defined domain of applicability, and (iv) appropriate measures of goodness of fit, robustness, and predictivity, and (v) possibly provide a mechanistic interpretation. The first criterion indicates that, as a given endpoint could be determined by different experimental protocols and under different experimental conditions, it is therefore crucial to precisely identify the experimental system that is being modeled by the QSAR [154]. The intent of having an unambiguous algorithm is to ensure transparency in the model algorithm that generates predictions of an endpoint. An applicability domain must be defined in the sense that QSARs are reductionist and thus are inevitably associated with limitations in terms of the types of chemical structures, physicochemical properties, and mechanisms of action for which the models can generate reliable predictions. The need for appropriate measures of goodness of fit, robustness, and predictivity indicates that the internal performance (as represented by the goodness of fit and robustness) and predictivity of a model (as determined by external validation) should be carefully assessed [154].

QSAR models have been developed in particular for metal and metal oxide nanomaterials [69,157,169]. Using a dataset of 17 metallic oxide particles differing by their chemical composition, it was found that their toxicity to *Escherichia coli* bacteria could be predicted on the basis of their chemical nature only thanks to calculated or experimental descriptors such as metal electronegativity and cation charge [155,170]. Surprisingly, dimensional or shape descriptors did not play any role. These results were confirmed in a model of mammalian cells, the HaCaT cell line, regarding the toxicity of 18 metal oxide nanoparticles [152,171]. More recently, QSAR models for the prediction of the inflammatory potential of metal oxide nanoparticles were developed based on a dataset of 30 samples [20]. Similarly, a QSAR model able to predict the cellular uptake of 109 functionalized metal oxide nanoparticles to pancreatic cancer cells (PaCa2) has been recently developed [172]. A predictive QSAR model for the cellular association of gold nanoparticles based on their physicochemical properties and protein corona fingerprints was developed and validated using a dataset of 105 nanoparticles [173]. 

Regarding the statistical methods used for QSAR models, different types of machine learning algorithms can be used (Figure 3), including principal component analysis (PCA), multiple linear regression (MLR), partial least squares (PLS) methods, random forests (RFs), support vector machines (SVMs), k-nearest neighbors, Bayesian networks, and artificial neural networks (ANNs) [20,149,166,172,174,175,176,177,178,179,180,181,182,183,184].

Despite the successful examples of application of nanoQSAR models, they still have some limitations. For instance, they did not prove very useful with regard to in vitro–in vivo extrapolation. Indeed, in QSAR models, the molecular descriptors usually refer to the pristine nanomaterials, while upon contact with biological media, these features are altered [69]. Similarly, because a nanomaterial can exert variable biological effects under different biochemical conditions (cell line, cell species, etc.), QSAR modeling is difficult [143]. Finally, it should be mentioned that one shortcoming of QSAR models lies in the fact that nanomaterial physicochemical features are studied independently while we know that they are directly correlated with each other (e.g., size and surface area). Such an assumption is, however, necessary to manage the complexity and large amount of data.

An alternative to QSAR, especially when data are significantly limited, is the read-across approach [152,165,185]. 

### 3.3. Grouping/Read-Across

To fill data gaps without performing additional experimental studies, the concepts of grouping and read-across have been developed. The aim is to predict the effects of an unknown substance (target material) based on the data available from substances classified in the same group because they are considered similar in some way (source material). Similarity is usually based on structural and/or physicochemical properties [13,145,150,152,165,186]. In other words, this approach is based on the assumption that materials that are considered similar may have a comparable toxicological behavior. Consequently, experimental available toxicological properties from a source material can be used to derive the toxicological properties of a target analogue with no (or limited) toxicological experimental data [187].

One may wonder if such an approach initially proposed for chemical compounds is suitable for nanomaterials due to their heterogeneity as previously discussed [13]. However, grouping/read-across methods are considered valid analytical tools to enable a more efficient hazard identification and assessment of nanomaterials by bringing together substances with similar hazardous profiles [145,146,186,188]. A major advantage of grouping/read-across methods is that they do not require a large amount of data to identify groups of similar compounds [152]. On the other hand, the criteria by which similar chemicals are selected for read-across should be carefully justified for the acceptability of the model predictions [183]. For this purpose, the European Chemicals Agency (ECHA) issued a guidance document on information requirements and chemical safety assessment specific to the application of QSAR and grouping approaches to nanomaterials [189]. 

As an example, following these guidelines, Lamon et al. used grouping/read-across to predict the in vitro comet assay results of TiO_2_ nanoforms. They identified the physicochemical properties that define the groups and the similarities between analogues of the same category, using chemoinformatic techniques such as hierarchical clustering (HC), principal component analysis (PCA), and random forest variable selection [190]. The level of similarity can also be determined with distance measures (Euclidean distance, Pearson correlation distance, Manhattan distance, etc.), and then the activity of the target nanomaterial(s) can be predicted based on the activity of the nearest neighbors (i.e., the most similar chemicals) [185]. Another way to justify (or reject) the grouping of a target nanomaterial and a source material is through integrated approaches to testing and assessment (IATAs) [145]. IATAs set out a tiered testing strategy, which reflects the different information needed and levels of uncertainty acceptable for different grouping purposes. The substantiation of a grouping decision is underpinned by the demonstration of similarity between group members, which helps the user to assess whether a target nanomaterial is sufficiently similar to a source material to allow grouping and to assume the target nanomaterial will induce similar toxicity to the source material [145].

Some examples from the literature illustrate the success of the grouping/read-across techniques. For instance, they allow estimating the cytotoxicity of metal oxide nanoparticles with a similar level of accuracy to that provided by QSAR models [152]. 

## 4. Conclusions

**Figure 4:**
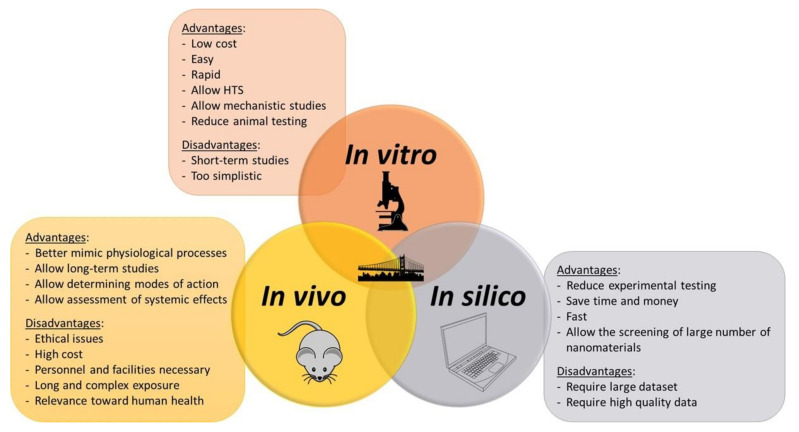
Summary of the advantages and limitations of approaches used in nanotoxicology and perspectives of bridging the gap between these methods.

As schematically summarized in Figure 4, in vitro/in vivo approaches as well as in silico models for nanotoxicology have their respective advantages and limitations.

No gold standard exists to assess the toxicity of a nanomaterial; instead, combinations of several techniques are necessary to adequately describe the material’s toxicological profile [55,56]. Similarly, it seems unlikely that one cell-based assay or computational model will ever replace an existing animal test. Thus, the most appropriate methodologies will need to be applied in an integrated assessment and testing strategy [69,191]. Experimental and computational approaches thus appear complementary, and their combination should lead to progress in the nanotoxicology field. Bridging the gap between these approaches implies stronger interactions between experimentalists and developers of models, adopting a multidisciplinary approach. In this context, new approach methodologies (NAMs) have been proposed; according to the ECHA definition, they consist of “a broad context to include in silico approaches, in chemico, and in vitro assays, as well as the inclusion of information from the exposure of chemicals in the context of risk assessment” [192]. As an example, system toxicology, by combining advanced analytical and computational tools, can provide quantitative information on system-wide molecular changes in the context of toxicant exposure, leading to information on how biological networks are perturbed by toxicants. System toxicology aims to change the way in which adverse effects of chemicals or other toxicants are characterized, from isolated empirical endpoints to integrated pathways of toxicity [112]. Similarly, the combination of omics analyses and machine learning has been proposed as a promising approach for analyzing nanotoxicity [139,154,184].

## Data Availability

Not applicable.
